# The association of vitamin D binding protein levels and genotypes with type 1 diabetes in the black South African population

**DOI:** 10.1186/s12902-022-01097-1

**Published:** 2022-07-17

**Authors:** Eleanor M Cave, Sureka Bhola, Nigel J Crowther, Carolyn J Padoa

**Affiliations:** 1 Department of Chemical Pathology, Faculty of Health Sciences, University of the Witwatersrand, Johannesburg, South Africa; 2 Department of Chemical Pathology, National Health Laboratory Service, Johannesburg, South Africa

**Keywords:** Type 1 diabetes, Black South African, Vitamin D binding protein

## Abstract

**Background:**

Vitamin D deficiency and the vitamin D pathway have previously been associated with type 1 diabetes (T1D). The majority of vitamin D is transported through the blood bound to the vitamin D binding protein (VDBP). Two polymorphisms in the *VDBP* gene (rs4588 and rs7041) result in different VDBP variants and have been associated with T1D, however the results are not consistent. The association of VDBP levels and its polymorphisms with T1D have not been investigated in the black South African population. Therefore, this study aimed to determine whether rs4588, rs7041 or serum VDBP levels were associated with T1D in this population.

**Methods:**

Participants with type 1 diabetes and controls were recruited from the greater Johannesburg area, South Africa. Participants were genotyped for rs4588 and rs7041 using PCR-RFLP and serum VDBP levels were determined by ELISA.

**Results:**

There was no difference in *VDBP* allelic or genotypic frequencies between participants with T1D and controls (rs4588 C allele frequency 0.92 vs. 0.94; *p* = 0.390 and rs7041 T allele frequency 0.95 vs. 0.95; *p* = 0.890). In univariate analysis, the rs4588 CC genotype was associated with increased serum VDBP levels, however, this association was lost with multivariate analysis. The *VDBP* genotypes were not associated with any other study variables. In logistic regression analysis, higher VBDP levels were associated with T1D (OR: (95% CI): 6.58 (1.45–29.9); *p* = 0.015), and within a linear regression analysis, T1D disease status was found to be associated with 0.044 mg/ml higher VDBP levels (*p* = 0.028).

**Conclusions:**

These data suggest that serum VDBP levels are positively associated with the presence of T1D in the African population. Whether VDBP lies in the causal pathway or its elevation is an effect of T1D is uncertain and requires further investigation.

## Background

Vitamin D has been shown to offer protection against type 1 diabetes (T1D) [1]. In order to exert its anti-inflammatory effects, vitamin D must be transported to the target tissue. The majority of vitamin D (approx. 85–90%) is transported in the circulation by the vitamin D binding protein (VDBP) [2, 3]. Vitamin D enters the cell via either passive diffusion of unbound vitamin D or endocytosis of VDBP-bound vitamin D. The endocytosis is mediated through the binding of VDBP to megalin and cubilin on the surface of cells [4, 5]. Thus, VDBP is thought to play a role in the development of T1D.

The VDBP molecule is a 58 kDa glycoprotein synthesized in the liver, coded for by the *Group-specific component* (*GC*: also known as *VDBP*) gene located on chromosome 4q11-q13 [6]. Two polymorphisms, rs7041 and rs4588, located in exon 11 of the *VDBP* gene result in different VDBP variants which have been shown to have different affinities for vitamin D [7, 8]. The rs7041 T > G polymorphism at position 416 of the *VDBP* gene results in a change from aspartic acid to glutamic acid (D432E), whereas the rs4588 C > A polymorphism, at position 420 results in a threonine to lysine (T436K) change [9]. Three common variants of the *VDBP* gene i.e. Gc1 fast (Gc1f; rs7041 T and rs4588 C alleles), Gc1 slow (Gc1s; rs7041 G and rs4588 C alleles) and Gc2 (rs7041 T and rs4588 A alleles), produce six possible VDBP phenotypes: Gc1f-1f, Gc1f-1s, Gc1f-2, Gc1s-1s, Gc1s-2 and Gc2-2 [10, 11]. The Gc1f variant has double the affinity for vitamin D than Gc1s and four times the affinity than Gc2 [12, 13]. In addition, Gc2-2 correlates with the largest free fraction of 25-hydroxyvitamin D and Gc1f-1f with the smallest [11]. The amount of VDBP-bound vitamin D that can enter target cells via endocytosis is known to correlate with the activation of the vitamin D receptor signalling pathway [14], thus, the polymorphisms may influence vitamin D signalling.

Ethnic differences in the genotype frequencies exist, with the Gc1f allele being more common in individuals of African ancestry and the Gc1s allele more common in Caucasians [15]. The Gc1 form is found at higher levels in the serum than Gc2, potentially due to differences in glycosylation patterns resulting in Gc2 being metabolised faster than Gc1 [16].

Few studies have looked at the association of these two *VDBP* gene polymorphisms with T1D. A study carried out on French Caucasians (110 individuals with T1D and 68 controls) found that the rs7041 G allele was associated with T1D [17]. However, the rs4588 polymorphism was not associated with the disease in this population [18]. In contrast, studies conducted in American white (203 individuals with T1D and 153 controls), Egyptian (59 individuals with T1D and 65 controls) and Turkish (55 individuals with T1D and 40 controls) populations failed to show an association between the two *VDBP* gene polymorphisms and T1D [19–21]. In addition, decreased levels of serum VDBP have been associated with T1D [19].

No studies have investigated differences in VDBP levels and the frequency of rs7041 and rs4855 polymorphisms in T1D in the South African black population, thus this study aimed to determine whether there is an association between these polymorphisms and serum VDBP levels and T1D in the black South African population.

## Methods

### Participant recruitment

Clinically diagnosed black South African participants with T1D (*n* = 169) were recruited from diabetes clinics in the greater Johannesburg area between October 2014 and September 2015. Clinical criteria (age at diagnosis < 30 years and initiation of insulin therapy within one year of diagnosis (therapy initiated immediately in children and adolescents)) were used to classify participants with T1D. Participants with an age at diagnosis > 30 years who required insulin treatment within the first 12 months post diagnosis and who were not obese were classified as having T1D. Black control participants that did not have diabetes (*n* = 165) were recruited from the South African Blood Transfusion Services blood drives and from students and staff based at the University of the Witwatersrand Medical School in Johannesburg. Participants were classified as controls if they declared that they were not on any medication to lower blood glucose levels and had a random blood glucose level below 11.1 mmol/L. Anthropometric measurements were taken, and a questionnaire completed for all participants. Patient glycated haemoglobin (HbA1c) and glucose levels were obtained from patient files. Participants who were pregnant, had obvious symptoms of an infection, clinical evidence of type 2 diabetes or secondary diabetes (i.e. from pancreatitis) were excluded from the study. Informed written consent was obtained from all participants prior to commencement of the study. The University of the Witwatersrand Human Research Ethics Committee (clearance certificate numbers M180334 and M150885) and the South African Blood Transfusion Services Research Ethics Committee (clearance certificate number 2014/19) approved the study protocol which conformed to the Declaration of Helsinki ethical guidelines.

### Measurement of glucose concentrations

Random plasma glucose concentrations for control participants were measured on the ADVIA Chemistry System (Siemens Health Care Diagnostics Inc., New York, USA) by the National Health Laboratory Services Chemical Pathology Diagnostic Laboratory based at the Charlotte Maxeke Johannesburg Academic Hospital. This system uses the enzymatic hexokinase method.

### Genoyping participants for the rs7041 and rs4588 *VDBP* polymorphisms

The DNA was extracted from 200 µl buffy coat using the Invisorb Spin Blood Mini DNA Extraction Kit (Invitek Molecular, Berlin, Germany) according to the manufacturer’s instructions. An 809 bp fragment of the *VDBP* gene flanking both polymorphisms was amplified using previously published primers (Forward: 5’CAAGTCTTATCACCATCCTG3’ and Reverse: 5’ GCCAAGTTACAATAACAC3’) [18]. Approximately 50ng of DNA was amplified using 2x KAPA Taq ReadyMix (Kapa Biosystems, Wilmington, MA, USA). Denaturation, annealing, and extension reactions were performed at 95 °C for 30 s, 57 °C for 30 s, and 72 °C for 30 s, respectively for 35 cycles in the BioRad T1000 Thermocycler (BioRad, Hercules, CA, USA). The resultant 809 bp PCR product was digested with StyI (rs4588) and HaeIII (rs7041) (New England Biolabs, Ipswich, MA, USA) for two hours at 37 °C in separate reactions. Participants were genotyped based on fragment sizes resolved on a 2% agarose gel. The rs4588 A allele introduces a StyI restriction site and results in fragments of 585 and 224 bp whereas the rs7041 G allele introduces a HaeIII restriction site generating fragments of 578 and 231 bp.

### Measurement of serum VDBP

Serum measurements of VDBP were determined using the Human Vitamin D Binding Protein ELISA kit (Abcam, Cambridge, United Kingdom) according to the manufacturer’s instructions. The intra- and inter-assay coefficients of variation for the assay were both < 10%.

### Statistical analysis

Normally distributed continuous variables were presented as mean ± standard deviation (SD) whilst skewed data were presented as median [lower quartile; upper quartile]. Categorical variables were presented as percentages (%) or frequencies. Glucose was log transformed to normality whereas the square root of T1D disease duration was used. No participants with T1D had either the rs4588 AA or the rs7041 GG *VDBP* genotypes and therefore the two-tailed Student’s non-paired t-test was used to compare variables between the remaining genotypes. An ANOVA was used to compare serum VDBP levels between the four seasons in which participants were recruited. A chi squared (χ^2^) test was used to compare all categorical variables. Multivariate linear regression analysis was performed with VDBP levels as the dependent variable and a multivariable logistic regression analysis was performed with T1D disease status as the dependent variable. Independent variables for both models were chosen based on scientific plausibility and their correlation (*p* < 0.20) with the dependent variable in univariate regression models. Type 1 diabetic subjects who were over the age of 30 years at diagnosis, of which there were three, were excluded from all analyses and the outputs were found not to change. Therefore, all analyses shown include these subjects. Results were considered statistically significant if the p-value was less than 0.05. Statistical analyses were performed using Statistica software version 13 (StatSoft, Tulsa, Oklahoma, USA).

## Results

### Clinical and phenotypic characteristics of patients with T1D and control participants

The clinical and phenotypic characteristics of the study participants are summarised in Table 1. Control participants were significantly older (*p* = 0.007), had a higher BMI (*p* < 0.001) and lower glucose concentration (*p* < 0.001) than participants with T1D.

**Table 1:** Clinical and phenotypic characteristics of the study participants

Variables	Cases (*n* = 169)	Controls (*n* = 165)	*p* value
Age (years)	26.4 ± 8.62	29.1 ± 9.45^a^	0.007
Age at diagnosis (year)	18.2 ± 6.92^b^	-	-
Duration of disease (years)	6.00 [2.00; 11.0]^b^	-	-
Sex			
Males, % (n)	50.9 (86)	44.2 (73)	0.224
Females, % (n)	49.1 (83)	55.8 (92)	
Glucose (mmol/L)	9.20 [5.75; 15.0]^c^	5.60 [4.65; 7.50]^d^	< 0.001
HbA1c (%)	10.7 ± 3.30^c^	-	-
BMI (kg/m^2^)	24.8 ± 5.89^e^	27.8 ± 6.01^f^	< 0.001
VDBP concentration (mg/mL)	0.43 ± 0.19^e^	0.41 ± 0.15^b^	0.356

Results are presented as median values [lower quartile; upper quartile] for skewed data and as mean ± standard deviation for non-skewed data and % (n) for categorical variables; Missing data: ^a^11, ^b^1, ^c^13, ^d^5, ^e^2, ^f^4

### Genotypic and allelic frequencies of rs4588 and rs7041

The rs4588 and rs7041 polymorphisms were both in Hardy-Weinberg equilibrium in our population (*p* = 0.431 and *p* = 0.222, respectively). There was no significant difference in genotypic or allelic frequencies for either polymorphism between cases and controls (Table 2).

**Table 2:** Allelic and genotypic frequencies of the rs4588 and the rs7041 polymorphisms in the study population

Polymorphism	Frequency (n)		*p* value
	**Cases**	**Controls**	
rs4588			
Genotypic model			
CC	0.84 (138)	0.90 (130)	0.571
CA	0.16 (27)	0.10 (18)	
AA	0.00 (0)	0.00 (0)	
Allelic model			
C	0.92 (303)	0.94 (278)	0.390
A	0.08 (27)	0.06 (18)	
rs7041 Genotypic model			
TT	0.91 (152)	0.91 (135)	0.990
TG	0.08 (13)	0.08 (12)	
GG	0.01 (1)	0.01 (1)	
Allelic model			
T	0.95 (317)	0.95 (282)	0.890
G	0.05 (15)	0.05 (14)	

### Association of *VDBP* genotypes with patient characteristics

Patient characteristics were compared between rs4588 (Table 3) and rs7041 (Table 4) genotypes. Participants with the rs4588 CC genotype had significantly higher VDBP levels compared to participants with the CA genotype (0.42 ± 0.17 vs. 0.37 ± 0.16 mg/mL; *p* = 0.038). However, no significant differences between rs7041 genotypes and participant characteristics were seen.

**Table 3:** Comparison of clinical characteristics according to the rs4588 genotypes in the total cohort

Variable	CC (*n* = 268)	CA (*n* = 45)	*p*-value
Age at diagnosis (years)g	18.0 ± 7.25^b^	18.7 ± 5.13	0.596
Duration of disease (years)g	5.00 [2.00; 11.0]^b^	8.00 [3.00; 13.0]	0.344
Sex			
Males, % (n)	48.1 (129)	51.1 (23)	
Females, % (n)	51.9 (139)	48.9 (22)	0.712
Glucose (mmol/L)	6.70 [4.90; 9.50]^c^	6.50 [4.70; 10.8]^b^	0.866
HbA1c (%)g	10.7 ± 3.42^d^	10.3 ± 2.77^e^	0.520
BMI (kg/m^2^)	26.0 ± 6.11^f^	27.0 ± 5.97^b^	0.323
VDBP concentration (mg/mL)	0.42 ± 0.17^e^	0.37 ± 0.16^b^	0.038

Results are presented as median values [lower quartile; upper quartile] for skewed data and as mean ± standard deviation for non-skewed data and % (n) for categorical variables; Missing data: ^a^9, ^b^1, ^c^17, ^d^10, ^e^2, ^f^5; ^g^calculated in participants with T1D only

**Table 4:** Comparison of clinical characteristics according to the rs7041 genotypes in the total cohort

Variable	TT (*n* = 287)	TG (*n* = 27)	*p*-value
Age at diagnosis (years)f	18.2 ± 7.08^b^	17.4 ± 4.96	0.702
Duration of disease (years)f	6.00 [2.00; 11.0]^b^	4.00 [2.00; 8.00]	0.451
Sex			
Males, % (n)	49.5 (142)	37.0 (10)	0.216
Females, % (n)	50.5 (145)	63.0 (17)	
Glucose (mmol/L)	6.80 [5.00; 9.80]^c^	5.40 [4.40; 9.60]^b^	0.844
HbA1c (%)f	10.6 ± 3.34^d^	11.6 ± 3.06^e^	0.315
BMI (kg/m^2^)	26.0 ± 5.89^a^	28.2 ± 7.76^b^	0.079
VDBP concentration (mg/mL)	0.42 ± 0.17^e^	0.39 ± 0.17^b^	0.374

Results are presented as median values [lower quartile; upper quartile] for skewed data and as mean ± standard deviation for non-skewed data and % (n) for categorical variables; Missing data: ^a^5, ^b^1, ^c^16, ^d^10, ^e^2; ^f^calculated in participants with T1D only

### Prevalence of VDBP binding affinity phenotypes and their associations with patient characteristics

The majority (77%) of the black South African cohort had a phenotype of 1f-1f whereas no participants with the 2–2 phenotype were identified (Table 5). There was no difference in the prevalence of binding affinity phenotypes (*p* = 0.615) between controls and participants with T1D. In addition, binding affinity phenotype was not associated with VDBP levels in our cohort (*p* = 0.072; data not shown).

**Table 5:** Percentage of binding affinity phenotypes in the study population

Binding affinity phenotype	Frequency (n)		
	**Cases (** ***n*** **= 165)**	**Controls (** ***n*** **= 148)**	**Total (** ***n*** **= 313)**
1f-1f	0.75 (124)	0.80 (118)	0.77 (242)
1f-1s	0.08 (13)	0.07 (11)	0.08 (24)
1f-2	0.16 (27)	0.12 (17)	0.14 (44)
1s-1s	0.01 (1)	0.01 (1)	0.01 (2)
1s-2	0.00 (0)	0.01 (1)	0.00 (1)
2–2	0.00 (0)	0.00 (0)	0.00 (0)

### The association of VDBP levels with season of sampling

The season in which participants were recruited were found to be significantly associated with VDBP levels (*p* < 0.001) with participants recruited in the autumn having the highest levels (0.48 ± 0.17 mg/mL) and those recruited in the spring having the lowest levels (0.36 ± 0.17 mg/mL) (Table 6). The same associations of VDBP levels with season were seen in both the control and the T1D cohort (both *p* < 0.001).

**Table 6:** VDBP levels in the total cohort according to season recruited

Season of sampling	Controls (n)	Control VDBP concentration (mg/mL)	Cases (n)	Patient VDBP concentration (mg/mL)	Combined VDBP concentration (mg/mL)
Summer	15	0.40 ± 0.12	35	0.38 ± 0.16	0.39 ± 0.15
Autumn	88	0.46 ± 0.15	47	0.52 ± 0.20	0.48 ± 0.17
Winter	1	0.44	28	0.42 ± 0.10	0.42 ± 0.10
Spring	60	0.35 ± 0.14	57	0.38 ± 0.20	0.36 ± 0.17

*p* < 0.001 for each of three ANOVAs for control, case and combined VDBP concentrations across seasons

### Determinants of T1D disease status

A logistic regression analysis was used to determine whether genotype or VDBP levels associated with T1D disease status after adjusting for possible confounding variables (Table 7). Thus, when controlling for sex and season of sampling, no association was seen between disease status and either genotype. However, higher VDBP levels were found to be significantly associated with T1D (*p* = 0.015) and sampling in autumn associated with a reduced odds ratio for T1D (*p* < 0.001).

**Table 7:** Logistic regression analysis of determinants of T1D disease status

Dependent variable	Independent variable	Odds ratio (95% CI)	*p* value
Patient/Control ^a^	VDBP concentration (mg/mL)	6.58 (1.45–29.9)	0.015
	Autumn^b^	0.31 (0.19–0.53)	< 0.001
	Sex^c^	1.15 (0.72–1.84)	0.566
	rs7041 genotype^d^	0.96 (0.41–2.25)	0.928
	rs4588 genotype^e^	0.71 (0.35–1.42)	0.332

For full model *p* < 0.001 (*n* = 310); variable coding: ^a^ patient = 1 and control = 0; ^b^autumn = 1 and not autumn = 0;^c^female = 1 and male = 0; ^d^TT = 1 and not TT = 0; ^e^CC = 1 and not CC = 0

### Determinants of VDBP levels

Multiple regression analysis was used to determine the variables which impact VDBP levels (Table 8). When controlling for sex, BMI, season recruited, VDBP genotype, age and disease status, having T1D, being recruited in autumn and female sex were associated with increased VDBP levels of 0.044, 0.112 and 0.041 mg/mL, respectively. This model accounted for 13.5% of the variance of VDBP levels.

**Table 8:** Multiple regression model for the determinants of VDBP levels

Dependent variable	Independent variable	B value	*p* value
VDBP concentration (mg/mL)	Patient/Control ^a^	0.044	0.028
	Autumn^b^	0.112	< 0.001
	Sex^c^	0.041	0.043
	rs7041 genotype^d^	0.016	0.646
	rs4588 genotype^e^	0.037	0.185
	Age (years)	-0.001	0.362
	BMI (kg/m^2^)	-0.001	0.491

Variable coding: ^ a^patient = 1 and control = 0; ^b^autumn = 1 and not autumn = 0; ^c^female = 1 and male = 0; ^d^TT = 1 and not TT = 0; ^e^CC = 1 and not CC = 0; for full model *p* < 0.001 (*n* = 296), *R*^2^ = 0.135

## Discussion

Polymorphisms in the *VDBP* gene and circulating VDBP levels have previously been associated with T1D, however, data is contradictory, and no studies have investigated these associations in the South African population. In our study, no association with T1D was found for either the VDBP gene polymorphisms (rs7041 and rs4588) or the VDBP affinity variants. The high binding affinity variants (1f-1f/1f-1s) had the highest frequency in both cases (0.83) and controls (0.87). In univariate analysis VDBP serum levels were significantly higher in participants with T1D with the rs4588 CC genotype compared to those with the CA genotype. However, in multivariate linear regression analysis, the genotypic associations with VDBP levels fell away and the presence of T1D, autumn and female sex were found to be positively associated with VDBP serum levels. In addition, VDBP serum levels were positively associated with risk for T1D in a multivariable logistic regression model. The reduced odds ratio of sampling in autumn for T1D is likely due to the fact that more controls than cases were recruited in this season (65.2 vs. 34.8%, respectively).

In our study, control participants were significantly older and had significantly higher BMI compared to participants with T1D. Several studies have investigated the association of age and BMI with VDBP levels. A study by Winters and colleagues [22] conducted in young adult African American and white women found that VDBP levels were unrelated to BMI. Similarly, a study on Iranian women showed no association between VDBP levels and BMI. In addition, no association was seen between VDBP levels and age [23]. Furthermore, age was not associated with VDBP levels in a cohort of participants with T1D [19]. The lack of association of BMI and age with VDBP levels was confirmed in our study by multiple regression analysis. These data therefore suggest that the older age and higher BMI of the control participants in our study would have little impact on the VDBP levels.

The association between *VDBP* genotypes (rs7041 and rs4588) and T1D has not consistently been shown in the literature. Ongagna and colleagues [17] found an association of the rs7041 G allele with T1D in a French population (110 participants with T1D and 68 controls), whereas Cooper and colleagues [24] found some evidence (*p* = 0.050) that rs4588 C > A polymorphism was associated with T1D in a British population (8 517 participants with T1D and 10 438 controls). However, studies in white American (181 participants with T1D and 163 controls), Egyptian (59 participants with T1D and 65 controls) and Turkish (55 participants with T1D and 40 controls) populations showed no association between T1D and either the rs7041 or rs4588 *VDBP* gene polymorphisms [20, 21, 25]. Similarly, our study did not find any association between rs4588 or rs7041 and T1D. In addition, no association was seen between frequency of binding affinity variants and T1D.

In our study, the presence of the rs4588 CC genotype was associated with higher serum VDBP levels when compared to the CA genotype in univariate analysis. This association was lost upon multiple regression analysis. No association was seen between rs7041 genotype and VDBP levels. A small study in a Turkish population found that the rs7041 GG genotype was associated with higher VDBP levels [21], whereas a study in 750 healthy American children found no association between VDBP levels and either the rs4588 or the rs7041 genotype [26]. Powe and colleagues [27] found the rs7041 G allele and the rs4588 A allele were associated with higher levels of VDBP in 3720 white and black adult Americans. Rivera-Paredez [28] found in a Mexican cohort (*n* = 1853) that both the rs4588 CC genotype and the rs7041 GG genotypes had significantly higher VDBP levels. Thus, these associations are not consistently seen. This may be due to the non-standardisation of VDBP measurement, ethnic differences, the season of sampling, influence of disease or the sample size.

Serum levels of VDBP were not found to be a determinant of T1D disease status in studies in American (115 participants with T1D and 55 controls), South Korean (42 participants with T1D and 29 controls) and Turkish populations (55 participants with T1D and 40 controls) [21, 29, 30]. A study by Blanton and colleagues [19] however, did show an association between lower levels of serum VBDP and T1D (203 participants with T1D and 153 controls). These studies are in contrast to our findings which showed that higher levels of VDBP were associated with T1D in a logistic regression model. These results may differ across studies due to sample sizes, different races being investigated and not controlling for season of sampling. No studies investigated newly diagnosed participants with the mean duration of disease ranging from 4 to 10 years. It is possible that VDBP plays a role in the aetiology of T1D through its immunomodulatory role. Thus, VDBP is known to enhance neutrophil chemoattractant activity [31] and can be converted to VDBP macrophage activating factor (VDBP-MAF) by sialidase and beta-galactosidase [32]. The VDBP may attract neutrophils to the pancreas, which then potentially result in pancreatic damage, potentially through laying down neutrophil extracellular traps (NETs), which have previously been found in the pancreas of patients with T1D [33]. The NETs activate trypsinogen which then amplifies the pancreatic injury [34]. In addition, VDBP-MAF may activate macrophages involved in the proinflammatory Th1 pathway which ultimately results in β-cell death [35].

The determinants of VDBP serum levels were investigated using multivariable linear regression analysis and participants recruited in autumn, female sex and those with T1D were found to be associated with higher VDBP levels. Season of sampling has previously been shown to affect VDBP levels [36]. Previous studies have shown that VDBP levels were associated with serum vitamin D levels albeit inconsistently [16, 37]. It is possible that as vitamin D is at its highest levels at the end of summer going into autumn [38] that this results in a corresponding increase in VDBP levels in the autumn. Higher oestradiol concentrations has previously been associated with higher VDBP levels [39], thus explaining the association of sex with VDBP levels. The presence of T1D was associated with higher VDBP levels in our study. We hypothesise that this is due to the inflammation associated with T1D [40]. An in vitro study by Guha et al. [41] found that pro-inflammatory cytokines such as IL-6 and TNF-α upregulated VDBP mRNA expression. Both IL-6 and TNF-α have been associated with chronic inflammation and the associated microvascular complications seen in participants with T1D [42–44]. Thus, the association between T1D and VDBP may be bi-directional, with VDBP increasing the risk of T1D, and T1D-associated inflammation then leading to higher serum levels of VDBP.

The limitations of the study were the medium sample size and the lack of vitamin D and cytokine measurements. In addition, the study was cross-sectional and therefore causation could not be investigated but only inferred from the statistical associations. Also, blood samples were collected over 12 months and it is known that there is a seasonal variation in VDBP serum levels [36], which was confirmed in our study. Ideally, the season of blood collection should have been matched between the groups to reduce confounding, however this was not possible in the current study. Therefore, the effect of season on VDBP levels was mitigated by adjusting for season of collection within the regression models. A further limitation of this study was that control participants were screened for the presence of diabetes using random blood glucose measurements only. Fasting plasma samples and HbA1c measurements were not available for this group. Lastly, differentiating between T1D and type 2 diabetes can be difficult, particularly in older subjects. This problem can be partially solved by measuring autoantibodies and/or serum C-peptide levels at the time of diagnosis. However, this data was not available for our study participants. Therefore, all T1D subjects in this study diagnosed at > 30 years-of-age were only included if they were not obese and all diabetics had to have started insulin therapy within 1 year of diagnosis, with all children and adolescents having had to start insulin therapy immediately at diagnosis. In addition, the T1D subjects diagnosed at > 30 years-of-age were excluded from all statistical analyses, but this was found to not alter the outputs. Although not ideal, we believe that these procedures will have substantially reduced the risk of the study data being compromised by the inclusion of misdiagnosed type 2 diabetic subjects.

## Conclusions

It is possible that a cyclical relationship exists between T1D and VDBP, with VDBP’s immunomodulatory properties increasing the risk for T1D and the inflammatory environment of T1D leading to enhanced levels of VDBP. The binding protein appears to affect T1D through its immunomodulatory properties where it may, in the early stages of T1D, be important in macrophage activation and attracting neutrophils to the pancreas causing tissue damage and ultimately β-cell death. Subsequently, due to chronic inflammation in the diseased state, the secretion of pro-inflammatory cytokines such as IL-6 and TNFα is increased, which enhance the transcription of VDBP. Within our cohort, there is poor glycaemic control (HbA1c: 10.7 ± 3.30%) and thus participants are more likely to have developed microvascular complications which have previously been shown to be associated with increased VDBP levels. Further research is required to confirm this hypothetical bidirectional relationship between T1D and VDBP.

## Data Availability

The dataset used and/or analysed during the current study is available from the corresponding author on reasonable request.
